# Treatment of periprosthetic femoral fractures after femoral revision using a long stem

**DOI:** 10.1186/s12891-015-0565-7

**Published:** 2015-05-10

**Authors:** Youngwoo Kim, Chiaki Tanaka, Hiroshi Tada, Hiroshi Kanoe, Takaaki Shirai

**Affiliations:** Department of Orthopaedic Surgery, Kyoto City Hospital, 1-2 Mibu,higashitakada-cho, Nakagyo, Kyoto 604-8845 Japan

**Keywords:** Total hip arthroplasty, Revision, Periprosthetic fracture, Long stem

## Abstract

**Background:**

Periprosthetic femoral fractures are becoming increasingly common and are a major complication of total hip arthroplasty and hemiarthroplasty. The treatment of periprosthetic femoral fracture after femoral revision using a long stem is more complex and challenging. The purpose of this study was to identify the clinical and radiographical features of periprosthetic femoral fractures after revision using a long stem.

**Methods:**

We report a retrospective review of the outcomes of treatment of 11 periprosthetic fractures after femoral revision using a long stem. Eleven female patients with a mean age of 79.2 years (70 to 91) were treated for a Vancouver type B1 fracture between 1998 and 2013. The mean numbers of previous surgeries were 3.1 (2 to 5).

**Results:**

The average follow-up was 58.9 months (8 to 180). We found several important features that might influence the outcome of treatment for periprosthetic femoral fractures after femoral revision using a long stem: 1) all cases were classified as Vancouver type B1. 2) 6 patients (55%) had a transverse fracture around the tip of the long stem. 3) 7 patients (64%) had a history of previous fracture of the ipsilateral femur. The type B1 fractures were treated with open reduction and internal fixation in 9 hips, 6 of which were reinforced with bone grafts. Two other periprosthetic fractures were treated with femoral revision. One was revised because of stem breakage, and the other was a transverse fracture associated with poor bone quality, which received a femoral revision with a long stem and a plate. All fractures except one achieved primary union. This failed case had a bone defect at the fracture site, and revision surgery using a cementless long stem and allografts was successful.

**Conclusions:**

These findings suggest that most cases of type B1 fracture after revision using a long stem have been treated successfully with open reduction and internal fixation. However, a transverse fracture with very poor bone quality might be considered as a type B3 fracture, and femoral revision might be a treatment of choice.

## Background

During the past decade, the number of patients requiring total hip arthroplasty (THA) has increased steadily in both younger patients and the more active elderly population [1]. There has been also a marked increase in hemiarthroplasty (HA) as treatment for femoral neck fractures [2]. Subsequently, periprosthetic femoral fractures are also becoming increasingly frequent and are a major complication of THA and HA [1,3,4]. A recent study showed that the incidence of periprosthetic femoral fracture is about 1% after primary THA and 4.2% after revision THA [5,6]. Periprosthetic fractures are also more common in patients who have sustained a hip fracture before arthroplasty [7]. The treatment of these fractures is complex and challenging, and requires the skills of both trauma surgery and revision arthroplasty [8]. The Vancouver classification developed by Duncan and Masri has become the universally accepted classification and has been validated recently in the European setting [9,10]. The site of the fracture, stability of the prosthesis, and the quality of bone stock are the three most important parameters used in the Vancouver classification to guide the surgeon in making a decision about the treatment of these fractures. Treatment is made more difficult by comorbid factors such as fragility or severe osteoporosis in elderly patients [11].

The risk factors for periprosthetic femoral fracture include poor bone stock, age, chronic use of corticosteroid, inflammatory arthropathy, stress risers, whether iatrogenic or caused by local osteolysis, previously surgery, excessively stiff joints, and various neurological conditions [1,6,12,13]. Because of the increase in the incidence of arthroplasty and life expectancy, revision arthroplasty is becoming more frequent. Long stem revision of the femoral component achieves immediate mechanical fixation by bypassing to the distal femoral bone stock in the case of aseptic loosening and periprosthetic fractures [14,15]. Orthopaedic surgeons are now frequently confronted with periprosthetic fractures in elderly patients after revision surgery using a long stem. The purpose of this study was to identify the clinical and radiographical features of periprosthetic femoral fractures after revision surgery using a long stem.

## Methods

We retrospectively reviewed a consecutive series of 11 periprosthetic femoral fractures that occurred after revision hip arthroplasty using a long stem between 1998 and 2013. The Institutional Review Board of Kyoto City Hospital approved the protocol for this study, and informed consent was obtained from each patient. The patients were all women with a mean age at the time of fracture of 79.2 years (70 to 91 years), as shown in Table 1. The status of the initial arthroplasty was THA in 5 patients and HA in 6 patients. The original diagnosis was femoral neck fracture in 5 patients, osteoarthritis in 5 patients, and avascular necrosis of femoral head in 1 patient. Seven patients had had a cemented femoral component and 4 had had a cementless femoral component. The mean numbers of previous surgeries were 3.1 (2 to 5). A previous history of fracture in the same femur was found in 7 hips including 5 femoral neck fractures, 3 periprosthetic fractures (Vancouver type B1: 1 hip, type B2: 2 hips). The status of the femur just before the latest revision was a cemented long stem in 2 patients, a cemented standard stem in four patients, a cementless standard stem in 3 patients and resection arthroplasty after infection in 2 patients. The cause of the latest revision surgery was aseptic loosening in 6 hips, periprosthetic fracture in 3 hips, and infection in 2 hips. The status of the femoral component after the latest revision was a cemented long stem in 8 patients, a cementless long stem in 2 patients, and a cemented standard stem with impaction bone grafting technique in 1 patient.

**Table 1:** **Demographic data**

**Case**	**Gender age**	**Original diagnosis**	**Primary treatment**	**Fixation**	**Previous operations**	**Previous fractures**	**Femoral status just before the latest revision**	**Revision diagnosis**	**Femoral component after the latest revision**
1	F 87	FNF	BHA	Cemented	3	FNF, SCF	Removal of the stem	Infection	Cemented long stem
2	F 73	OA	THA	Cemented	3	PPFx. B1	Cemented long stem	PPFx. B1	Cemented long stem
3	F 73	FNF	BHA	Cemented	5	FNF	Cemented long stem	Aseptic lossening	Cemented standard stem
4	F 73	OA	THA	Cemented	3	PPFx. B2	Cemented standard stem	PPFx. B2	Cementless long stem
5	F 70	OA	THA	Cemented	3	-	Cemented standard stem	Aseptic lossening	Cemented long stem
6	F 79	OA	THA	Cemented	2	-	Cemented standard stem	Aseptic lossening	Cemented long stem
7	F 71	ANF	BHA	Cementless	5	-	Removal of the stem	Infection	Cemented long stem
8	F 81	FNF	BHA	Cementless	3	FNF, SCF	Cementless standard stem	Aseptic lossening	Cemented long stem
9	F 91	OA	THA	Cemented	2	-	Cementless standard stem	Aseptic lossening	Cemented long stem
10	F 82	FNF	BHA	Cementless	2	FNF, SCF	Cementless standard stem	Aseptic lossening	Cemented long stem
11	F 91	FNF	BHA	Cementless	3	FNF, PPFx. B2	Cementless standard stem	PPFx. B2	Cementless long stem

FNF, femoral neck fracture; OA, osteoarthritis; ANF, avascular necrosis of femoral head; BHA, bipolar hemiarthroplasty; THA, total hip, arthroplasty; PPF, periprosthetic femoral fracture; SCF, suprachondylar fracture.

Nine patients had sustained a clear mechanical fall, and 2 had sustained a spontaneous or insufficiency fracture. The fractures were classified according to the Vancouver classification. The management algorithm of the Vancouver classification system was generally applied, but it was modified in some cases according to the surgeon’s decision and the operative field. We have added bone grafting in cases of transverse fracture or short oblique fracture with comminution. The bone quality of these fractures was assessed using cortical index [16]. Gruen have reported that the mean value of cortical index was 50.9% in patients with degenerative joint disease and 43.9% in patients with femoral neck fractures. All fractures were treated surgically. Patients were followed up clinically and radiographically for 6 weeks, and 3, 6, and 12 months postoperatively, and annually thereafter.

The time to union was recorded. Clinical union was defined as full weight bearing with only slight or no occasional pain that did not compromise walking or basic daily activities. Union was defined as clinical union in the presence of radiological evidence of bone bridging in both the anteroposterior and lateral views. At the final follow-up, clinical evaluations were performed using the Japanese Orthopedic Association (JOA) score [17]. Complications were recorded with particular reference to infection, refracture, dislocation, and loosening. Postoperative rehabilitation was specific to each patient, but active flexion and extension of the ankle, hip, and knee of the affected side on the first day were common to all. Weight bearing was usually allowed when the fracture callus was visible radiologically and the application of weight was pain free. After 6 weeks, patients were allowed non-weight-bearing movement with a frame or crutch until early signs of healing and then advanced to partial weight bearing at 8 to 12 weeks postoperatively. Once union was confirmed, full weigh bearing was started about 3 to 4 months after surgery. All operations were performed by two experienced senior surgeons (CT and YK).

## Results

**Figure 1:**
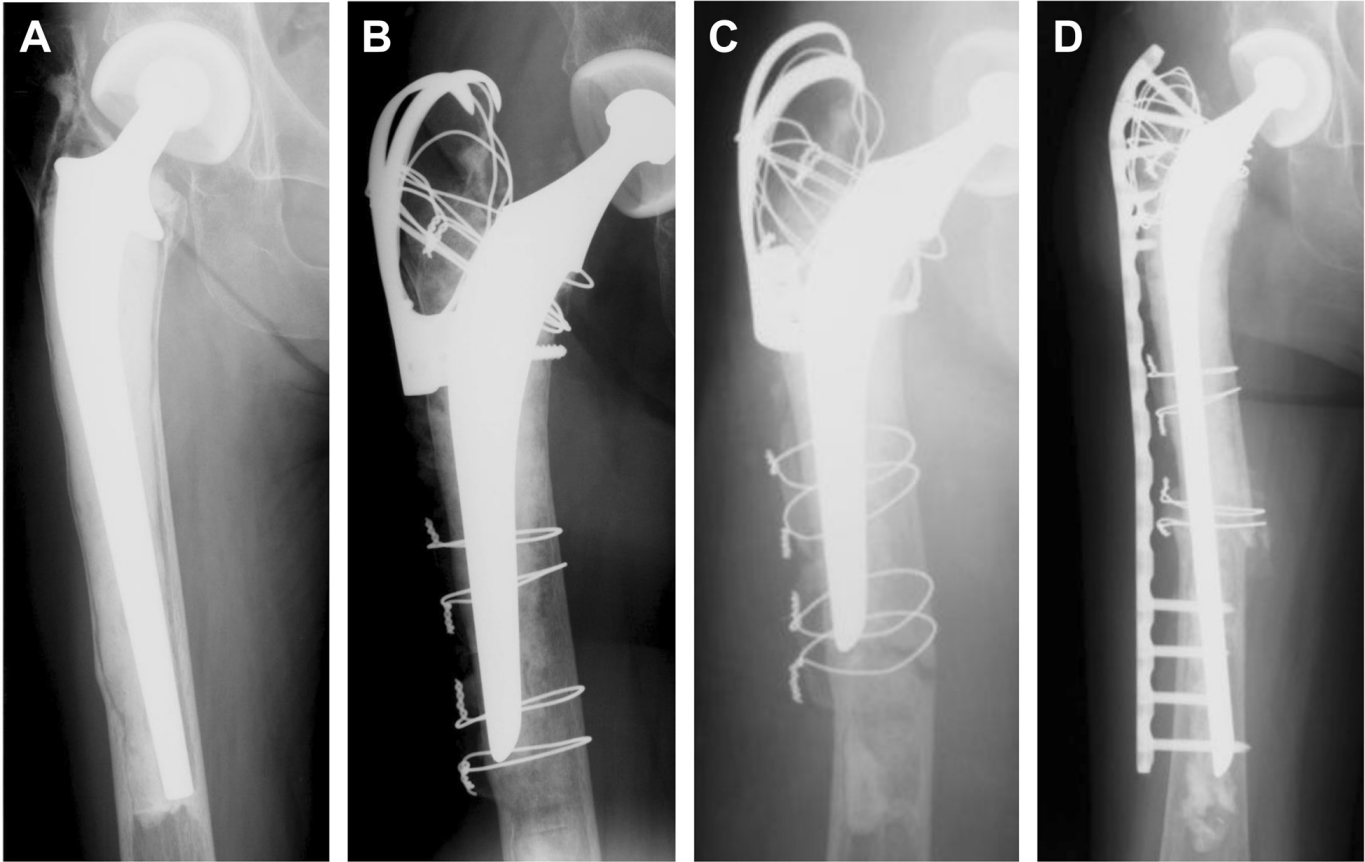
Case 3, A seventy-three-years-old woman who sustained a Vancouver type-B1 periprosthetic femoral fracture seventy five months after revision surgery using a long stem. **A** Anteroposterior radiograph of the right hip showing the loosening of long stem. **B** Anteroposterior radiograph of the right hip made just after revision surgery using a standard stem with impaction bone grafting. **C** Anteroposterior radiograph of the right hip showing the Vancouver type B1 fracture pattern six months after revision surgery. **D** Anteroposterior radiograph of the right hip made one year after operation. Incorporation of the fracture site can be observed.

The mean time of the radiographic and clinical follow-up was 58.9 months (8 to 180 months). All fractures were classified as Vancouver type B1. The fracture pattern was a transverse fracture in 6 hips and an oblique fracture in 5 hips (Table 2). The mean time from the revision arthroplasty using a long stem to the fracture was 106.5 months (12 to 240 months). The mean cortical index was 20.5% (13.8 to 34.0%). In this study, the type B1 fractures were fixed with a plate in 9 patients. Seven patients were treated with a locking compression plate and cerclage wiring and, 2 patients were treated with a Dall–Miles system (Howmedica, Rutherford, NJ). Six patients were treated with bone allografts or autografts to facilitate fracture healing. In the other 2 patients, revision arthroplasty was performed with a cemented long stem using an impaction bone grafting technique. In one of them, femoral revision was necessary because of stem breakage (Case 4). In this case, cortical index was 15.3%. Another patient required reinforcement with a locking compression plate fixation (Case3, Figure 1). This patient was a 73-year-old female who had undergone revision surgery using impaction bone grafting because of aseptic loosening of the long stem after hemiarthroplasty. Six months after revision, this patient sustained a periprosthetic femoral fracture and had required revision arthroplasty using long stem because of very poor bone quality (cortical index; 13.8%) and had required reinforcement with a locking compression plate fixation because the length of the long stem available seemed to be insufficient and the reduction of the fracture was not ideal (in valgus alignment).

**Table 2:** **Results**

**Case**	**Fracture type**	**Time to fracture (mo)**	**Treatment**	**Plates**	**Bone graft**	**Time to union (mo)**	**Duration of follow-up (mo)**	**Outcome**	**JOA score**
1	Oblique	104	ORIF	DFLCP	-	4	22	Union	63
2	Transverse	100	ORIF	Reverse DFLCP	Auto, fibula	6	28	Union	77
3	Transverse	75	Revision (IBG) +ORIF	LCP	Allo, auto, iliac	3	119	Union	68
4	Transverse (with stem fracture)	88	Revision (IBG)	-	Allo	4	45	Union	77
5	Transverse	78	ORIF	Dall-Miles plate	Auto, fibula	7	115	Nonunion*	53
6	Oblique	99	ORIF	LCF	Auto, iliac	4	74	Union	60
7	Transverse	12	ORIF	Dall-Miles plate	Auto, fibula	8	180	Union	67
8	Oblique	120	ORIF	DFLCP	Allo	4	35	Union	60
9	Oblique	142	ORIF	DFLCP	-	4	10	Union	62
10	Transverse	114	ORIF	Reverse DFLCP	Auto, iliac	6	12	Union	72
11	Oblique	240	ORIF	DFLCP	-	2	8	Union	81

*Breakage of the plate; ORIF, open reduction and internal fixation; IBG, impaction bone grafting; DFLCP, distal femur locking compression plate; LCP, locking compression plate; auto, autograft; allo, allograft.

**Figure 2:**
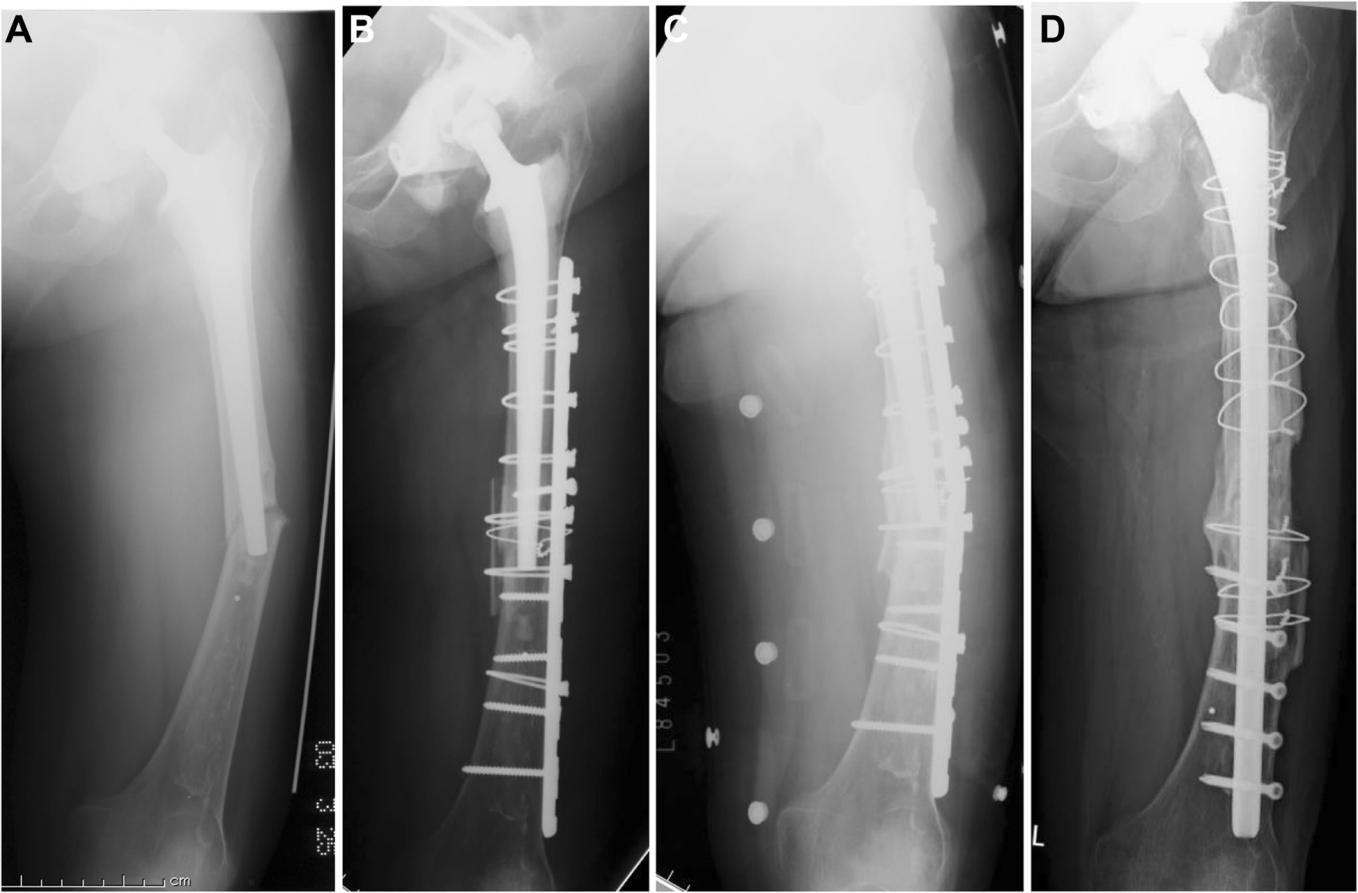
Case 5, A seventy- years-old woman who sustained a Vancouver type-B1 periprosthetic femoral fracture seventy eight months after revision surgery using a long stem. **A** Anteroposterior radiograph of the left hip showing the Vancouver type-B1 frcture pattern. **B** Anteroposterior radiograph of the left hip made just after operation. **C** Anteroposterior radiograph of the left hip showing the nonunion with breakage of the plate. **D** Anteroposterior radiograph of the left hip made one year after operation. Incorporation of the fracture site can be observed.

In 1 patient treated with a Dall–Miles cable plate and autogarfts, failure occurred because of plate breakage (Case 5, Figure 2). This case was revised with a cementless long stem (Cannulok revision prosthesis; Orthodynamics, Christchurch, Dorset, UK) in addition to allogeneic tibial plates, which resulted finally in bone union. Union occurred in all patients except one without malunion or infection. This failed case with breakage of the plate had a bone defect at the fracture site, and revision surgery using a cementless long stem and allografts was successful. The mean time to weight bearing for these patients was 8 weeks. Complete fracture union occurred in all patients at an average of 5 months (2 to 8 months). None of the implants were radiolographically loose at final follow-up. The final mean functional score of the patients was JOA hip score 67.2.

## Discussion

Our study is among the first to report on the clinical and radiographic assessment of periprosthetic femoral fractures after femoral revision using a long stem. Most studies on periprosthetic femoral fractures deal with standard cemented or cementless stems [18-20]. We think that there is a slight difference between periprosthetic fractures with a standard stem and periprosthetic fractures after revision surgery using a long stem. We found several important features that might influence the outcome of treatment for periprosthetic femoral fractures after femoral revision using a long stem.

First, all cases in our series were classified as Vancouver type B1. In general, Vancouver type B1 fracture should be treated with open reduction and internal fixation. However, good fixation was less easily achieved in the presence of osteoporosis, which is common in this group of patients [11].

Second, 6 patients (55%) had a transverse fracture around the tip of the long stem. The rate of transverse fracture in our series is higher than that reported by other recent series [18]. Recent study of short oblique or transverse Vancouver type B1 fracture reported less satisfactory results and concluded that locking plate alone are insufficient for the treatment of periprosthetic femoral fracture and should be supplemented with cortical strut grafts [21].

Third, in our series, 7 patients (64%) had a history of previous fracture of the ipsilateral femur. Five femoral neck fractures, 3 periprosthetic fractures, and 3 suprachondylar fractures were included. The age at the index operation was 79.2 years, and previous surgeries were performed 3.1 on average. These facts mean that most patients had poor bone quality because of osteoporosis and previous surgeries.

**Figure 3:**
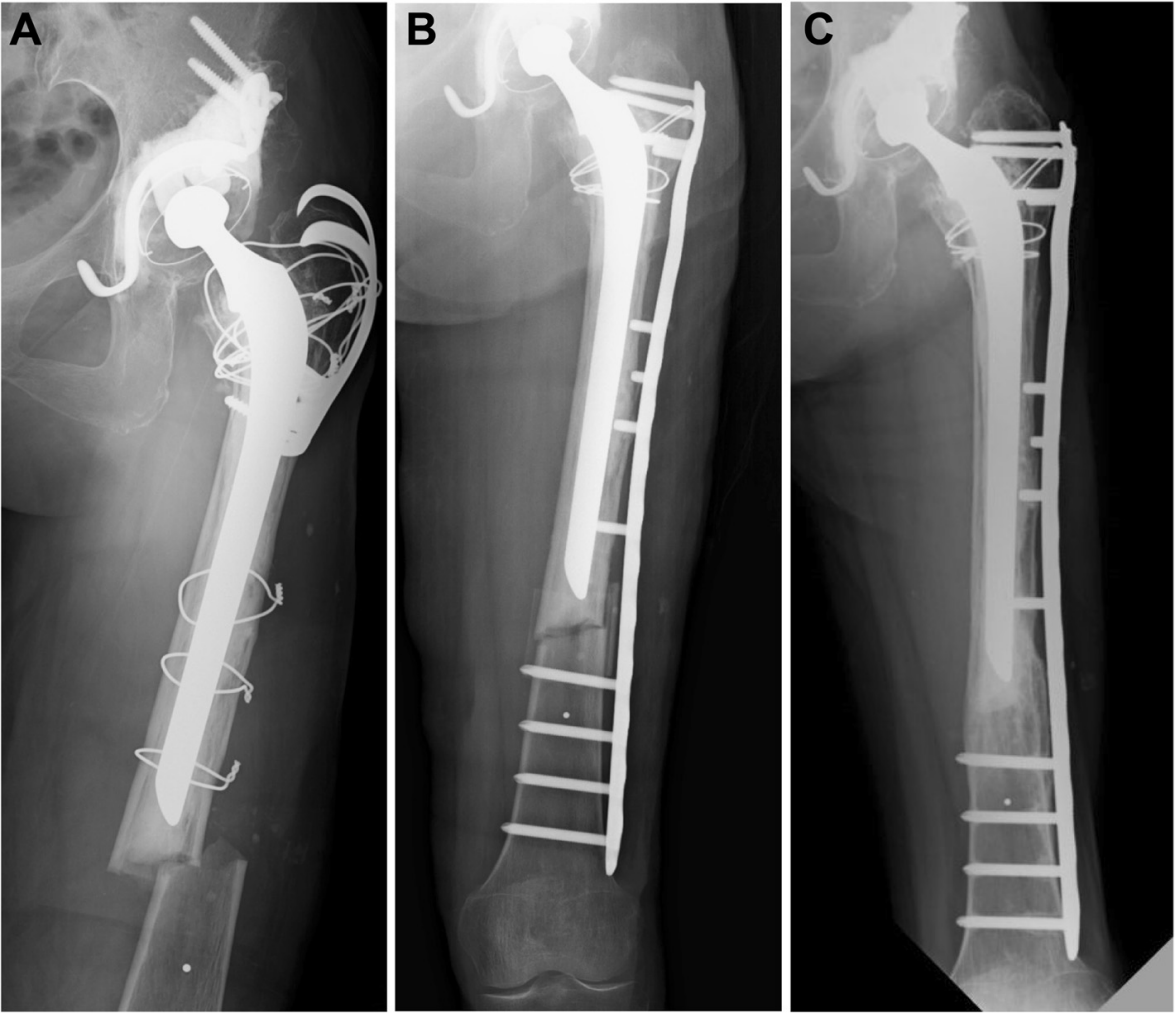
Case 2, A seventy-three-years-old woman who sustained a Vancouver type-B1 periprosthetic femoral fracture one handred months after revision surgery using a long stem. **A** Anteroposterior radiograph of the left hip showing the Vancouver type-B1 frcture pattern. **B** Anteroposterior radiograph of the left hip made just after operation. **C** Anteroposterior radiograph of the left hip made one year after operation. Incorporation of the fracture site can be observed.

According to the Vancouver classification, type B1 fractures are recommended to be treated with open reduction and internal fixation (ORIF) without revision of the femoral component. In our patients, 9 periprosthetic femoral fractures were treated with ORIF using conventional or locking compression plate and cerclage wiring with or without bone grafts. Five of the 9 fractures were oblique fractures and were treated successfully with this method. Bone grafting was performed in 2 because they involved oblique fracture with comminution. Four of 9 fractures were transverse fractures. Bone grafting was associated with ORIF because a stable long stem increases the stress at the distal end, which can make bone union very difficult. In 3 patients, the bone united without problems as shown in Figure 3, but in 1 of these patients, bone union failed because of breakage of the plate (Figure 2). This failure was associated with a bone defect at the fracture site. The high stress concentration at the end of the stable long stem combined with this bone defect might have caused the failure despite the bone grafting. Revision surgery was performed with a cementless long stem in addition to allogeneic tibial bone plates with success.

Two other periprosthetic fractures were transverse fractures and were treated by femoral revision. Femoral revision was necessary for 1 fracture because of stem breakage and for the other because of very poor bone quality (cortical index; 13.8%). This latter case had required revision previously with impaction bone grafting and conversion of the long stem to a standard stem because of loosening of the long cemented stem. Six months after this revision, the transverse fracture occurred at the zone with very poor bone quality. Femoral revision was performed using a long cemented stem, bone grafts, and a locking plate. This fracture united 6 months after the femoral revision.

In our patients, 7 periprosthetic fractures were treated with locking compression plates, and all patients achieved union. Recent studies have shown that locking compression plates represent a valuable advance in fracture treatment because they provide angular stability and stiffness in axial loading and torsion, help preserve the vascular supply, allow for the possible use of minimally invasive insertion techniques, require less plate-contouring compared with conventional plates, and provide better stability in osteoporotic bone [22-28]. Bi- or unicortical screw fixation at the level of the femoral shaft is effective against rotational stress. We used a reverse anatomic distal femoral locking compression plate, which involves use of an upside-down distal femoral plate of the contralateral side. The reverse use of the plate was intended to achieve multiple proximal trochanteric bicortical screw fixation [29]. This technique was very effective. Our results also show that plate pullout was not observed because cerclage wiring had been used routinely. Several authors have reported that plate fixation without cerclage wiring failed in 21% of patients because of plate pullout [21].

Usually bone grafting is not considered necessary for type B1 fractures if the fixation is stable. However, the authors recommend bone grafting for fractures after femoral revision using a long stem in cases involving transverse fracture or the short oblique fracture with comminution to improve fracture healing and increase bone stock. Stress concentration at the end of the stable stem seems to be much higher that of the standard stem [30,31]. And the bone quality is mostly poor because of multiple previous surgeries. Furthermore the patients in our series had a greater degree of osteoporosis (mean cortical index; 20.5%) than a matched group whose primary surgery had been performed for osteoarthritis (11hips, female n = 11, mean age; 78.1, mean cortical index; 53.7%). These aspects make bone union by ORIF more difficult. A transverse fracture with very poor bone quality (cortical index < 20) or bone loss might be considered as a type B3 fracture, and femoral revision might be the treatment of choice.

Some authors have reported that stem revision for transverse type B1 fractures is considered because this fracture configuration is difficult to control with single plating, and fixation with a long stem is necessary to achieve axial stability and healing [19,25]. In addition, impaction bone grafting with long stem can be used successfully for treatment of type B2 and B3 periprposthetic femoral fracture [15].

There were several limitations to this study. First, this study was retrospective design and the lack of a patient control group for comparison. Secondly, the series was too small and the follow up period was too short. Thirdly, the strategies of treatment for fractures were heterogeneous (e.g., Dall-miles system, locking plate, cementless long stem, and cemented long stem).

## Conclusions

In conclusion ORIF is the first choice of treatment for a type B1 fracture after revision using a long stem. If it is associated with poor bone quality ORIF also should be recommended in combination with bone grafts especially for transverse B1 fractures. However, a transverse fracture with very poor bone quality or bone loss might be considered as a type B3 fracture, and femoral revision might be the treatment of choice. The long-term effects and complications should be examined to determine whether these results can be maintained over time.
